# Crystal structure of 2-(4-meth­oxy­phen­yl)-6-nitro­imidazo[1,2-*a*]pyridine-3-carbaldehyde

**DOI:** 10.1107/S2056989015021957

**Published:** 2015-11-21

**Authors:** Mohamed Koudad, Abdelmalik Elaatiaoui, Noureddine Benchat, Mohamed Saadi, Lahcen El Ammari

**Affiliations:** a Laboratoire de Chimie Appliquée et Environnement (LCAE), Faculté des Sciences, Université Mohammed Premier, BP 524, 60000-Oujda, Morocco; b Observatoire de la Lagune Marchica de Nador et Région Limitrophe, Université Mohammed Premier, Faculté Pluridisciplinaire de Nador, BP 300, Selouane 62702 Nador, Morocco; c Laboratoire de Chimie du Solide Appliquée, Faculté des Sciences, Université Mohammed V, Avenue Ibn Battouta, BP 1014, Rabat, Morocco

**Keywords:** crystal structure, imidazo[1,2-*a*]pyridine, carbaldehyde, hydrogen bonding

## Abstract

In the title compound, C_15_H_11_N_3_O_4_, the imidazo[1,2-*a*] pyridine ring system is almost planar [r.m.s. deviation = 0.028 (2) Å]. Its mean plane makes dihedral angles of 33.92 (7) and 34.56 (6)° with the meth­oxy­phenyl ring and the nitro group, respectively. The cohesion of the crystal structure is ensured by C—H⋯N and C—H⋯O hydrogen bonds, forming layers almost parallel to the *ac* plane.

## Related literature   

For biological activities of derivatives of the title compound, see: Rupert *et al.* (2003 ▸); Hranjec *et al.* (2007 ▸); Hamdouchi *et al.* (1999 ▸); Rival *et al.* (1992 ▸); Scribner *et al.* (2008 ▸); Bode *et al.* (2011 ▸). For the synthesis of similar compounds, see: Sumalatha *et al.* (2009 ▸); Elaatiaoui *et al.* (2014 ▸, 2015 ▸).
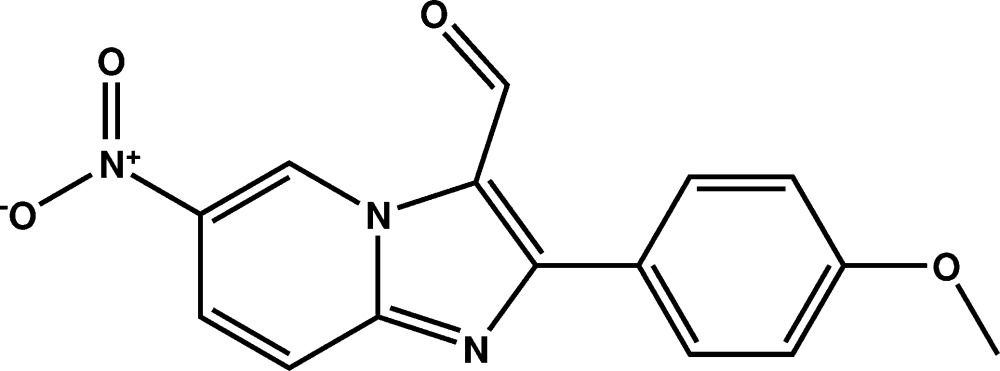


## Experimental   

### Crystal data  

C_15_H_11_N_3_O_4_
*M* *_r_* = 297.27
Monoclinic, 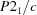

*a* = 10.8516 (6) Å
*b* = 12.0710 (6) Å
*c* = 10.2631 (5) Å
β = 93.200 (2)°
*V* = 1342.26 (12) Å^3^
*Z* = 4
Mo *K*α radiation
μ = 0.11 mm^−1^
*T* = 296 K
0.42 × 0.31 × 0.26 mm

### Data collection  

Bruker X8 APEX diffractometer
Absorption correction: multi-scan (*SADABS*; Bruker, 2009 ▸) *T*_min_ = 0.673, *T*_max_ = 0.746
25892 measured reflections
3472 independent reflections
2139 reflections with *I* > 2σ(*I*)
*R*_int_ = 0.060

### Refinement  

*R*[*F* ^2^ > 2σ(*F* ^2^)] = 0.044
*wR*(*F* ^2^) = 0.120
*S* = 1.02
3472 reflections
200 parameters
H-atom parameters constrained
Δρ_max_ = 0.21 e Å^−3^
Δρ_min_ = −0.16 e Å^−3^

### 

Data collection: *APEX2* (Bruker, 2009 ▸); cell refinement: *SAINT* (Bruker, 2009 ▸); data reduction: *SAINT*; program(s) used to solve structure: *SHELXS97* (Sheldrick, 2008 ▸); program(s) used to refine structure: *SHELXL2014* (Sheldrick, 2015 ▸); molecular graphics: *ORTEP-3 for Windows* (Farrugia, 2012 ▸); software used to prepare material for publication: *PLATON* (Spek, 2009 ▸) and *publCIF* (Westrip, 2010 ▸).

## Supplementary Material

Crystal structure: contains datablock(s) I. DOI: 10.1107/S2056989015021957/su5244sup1.cif

Structure factors: contains datablock(s) I. DOI: 10.1107/S2056989015021957/su5244Isup2.hkl

Supporting information file. DOI: 10.1107/S2056989015021957/su5244Isup3.cml

**Fig. 1.**
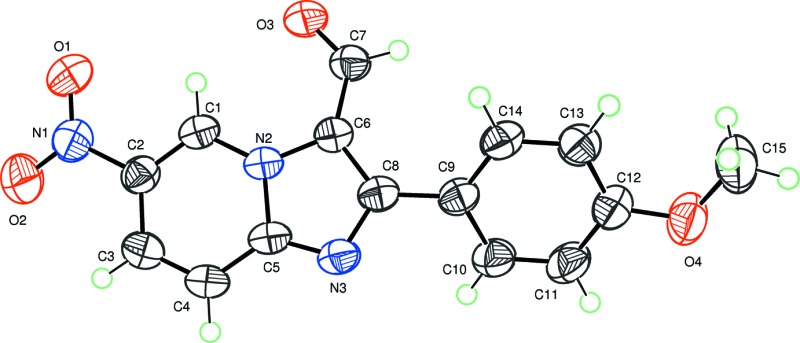
A view of the molecular structure of the title compound, with atom labelling. Displacement ellipsoids are drawn at the 50% probability level.

. DOI: 10.1107/S2056989015021957/su5244fig1.tif
A view of the mol­ecular structure of the title compound, with atom labelling. Displacement ellipsoids are drawn at the 50% probability level.

**Fig. 2.**
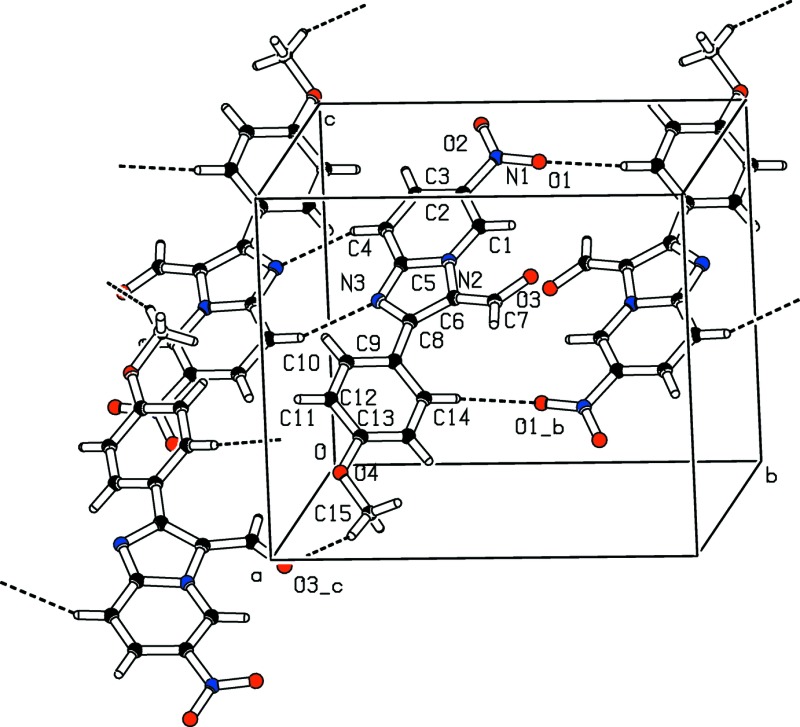
A partial view of the crystal packing of the title compound, showing molecules linked by hydrogen bonds (dashed lines; Table 1), forming layers parallel to (101).

. DOI: 10.1107/S2056989015021957/su5244fig2.tif
A partial view of the crystal packing of the title compound, showing mol­ecules linked by hydrogen bonds (dashed lines; Table 1), forming layers parallel to (101).

CCDC reference: 1437519

Additional supporting information:  crystallographic information; 3D view; checkCIF report

## Figures and Tables

**Table 1:** Hydrogen-bond geometry (Å, °)

*D*—H⋯*A*	*D*—H	H⋯*A*	*D*⋯*A*	*D*—H⋯*A*
C4—H4⋯N3^i^	0.93	2.53	3.412 (2)	158
C15—H15*A*⋯O3^ii^	0.96	2.52	3.423 (2)	158
C14—H14⋯O1^iii^	0.93	2.50	3.425 (2)	177

Symmetry codes: (i) 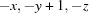; (ii) 
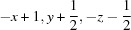
; (iii) 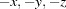.

## supplementary crystallographic information

### S1. Structural commentary

Imidazo[1,2-*a*]pyridines are a very inter­esting class of heterocyclic compounds because of their wide use in medicinal chemistry for the production of various pharmacologically active agents. In addition, various derivatives of imidazo[1,2-*a*]pyridine show a wide range of biological activities such as anti-inflammatory (Rupert *et al.*, 2003), anti­tumor (Hranjec *et al.*, 2007), anti­viral (Hamdouchi *et al.*, 1999), anti­bacterial (Rival *et al.*, 1992), anti­parasitic (Scribner *et al.*, 2008) and anti-HIV (Bode *et al.*, 2011). The access to the imidazo[1,2-*a*]pyridine scaffold is generally made by condensation of 2-amino­pyridine with a specifically chosen α-halo ketone (Sumalatha *et al.*, 2009). The present paper is a continuation of our research devoted to the development of imidazo[1,2-*a*]pyridines derivatives with potential pharmacological activities (Elaatiaoui *et al.*, 2014;2015).

In the title compound, Fig. 1, the fused five- and six-membered rings of the imidazo[1,2-*a*]pyridine moiety are virtually coplanar, with a maximum deviation of 0.028 (2) Å for atom C3. Its mean plane makes dihedral angles of 33.92 (7) and 34.56 (6)° with the meth­oxy­phenyl ring and the nitro group, respectively.

In the crystal, molecules are linked by C4–H4···N3, C15–H15A···O3 and C14–H14···O1 hydrogen bonds (Table 1), building layers parallel to (101), as shown in Fig. 2.

### S2. Synthesis and crystallization

Phospho­rus oxychloride (3.06 g, 0.02 mol) was added to a solution of 2-(4-meth­oxy­phenyl)-5-nitro­imidazo[1,2-*a*]pyridine (2.69 g, 0.01 mol) in DMF (25 ml) at room temperature under stirring. The mixture was heated to 353 K for 5 h. The resulting solution was evaporated to dryness *in vacuo*, and the residue was treated with cold water, filtered, and crystallized from methanol to give pure product (yield 74%, Rf = 0.45 (silica, CH_2_Cl_2_/MeOH, 9/1).

Spectral data: ^1^H NMR (300 MHz, DMSO, δ(p.p.m.)): 10.40 (s, 1H, C_5_H); 10.09 (s, 1H, CH=O); 8.36 (dd, 1H, C_7_H, J=2.4 Hz); 7.96 (m, 3H,*C*_8_H, CphH, CphH, J= 28.2 Hz); 7.10 (d, 2H, CphH, CphH, J=9 Hz); 3.83 (s, 3H, OCH_3_). ^13^C NMR (75 MHz, DMSO) δ(p.p.m.): 180.84; 161.64; 158.81; 147.76; 138.75; 131.75; 128.32; 125.06; 124.05; 121.11; 117.04; 115.02; 55.87. m/*z* (*M*+1): 298.09.

### S3. Refinement

Crystal data, data collection and structure refinement details are summarized in Table 1. H atoms were located in a difference Fourier map and treated as riding: C–H = 0.93-0.96 Å with *U*_iso_(H) = 1.5 *U*_eq_ for methyl H atoms and 1.2 *U*_eq_ for other H atoms. The reflection (100) affected by the beam-stop was removed during refinement.

### Crystal data

**Table:**

C_15_H_11_N_3_O_4_	*F*(000) = 616
*M**_r_* = 297.27	*D*_x_ = 1.471 Mg m^−^^3^
Monoclinic, *P*2_1_/*c*	Mo *K*α radiation, λ = 0.71073 Å
*a* = 10.8516 (6) Å	Cell parameters from 3472 reflections
*b* = 12.0710 (6) Å	θ = 2.5–28.7°
*c* = 10.2631 (5) Å	µ = 0.11 mm^−^^1^
β = 93.200 (2)°	*T* = 296 K
*V* = 1342.26 (12) Å^3^	Block, colourless
*Z* = 4	0.42 × 0.31 × 0.26 mm

### Data collection

**Table:**

Bruker X8 APEX diffractometer	3472 independent reflections
Radiation source: fine-focus sealed tube	2139 reflections with *I* > 2σ(*I*)
Graphite monochromator	*R*_int_ = 0.060
φ and ω scans	θ_max_ = 28.7°, θ_min_ = 2.5°
Absorption correction: multi-scan (*SADABS*; Bruker, 2009)	*h* = −14→14
*T*_min_ = 0.673, *T*_max_ = 0.746	*k* = −16→16
25892 measured reflections	*l* = −13→10

### Refinement

**Table:**

Refinement on *F*^2^	Hydrogen site location: inferred from neighbouring sites
Least-squares matrix: full	H-atom parameters constrained
*R*[*F*^2^ > 2σ(*F*^2^)] = 0.044	*w* = 1/[σ^2^(*F*_o_^2^) + (0.0468*P*)^2^ + 0.3011*P*] where *P* = (*F*_o_^2^ + 2*F*_c_^2^)/3
*wR*(*F*^2^) = 0.120	(Δ/σ)_max_ < 0.001
*S* = 1.02	Δρ_max_ = 0.21 e Å^−^^3^
3472 reflections	Δρ_min_ = −0.16 e Å^−^^3^
200 parameters	Extinction correction: *SHELXL2014* (Sheldrick, 2015), Fc^*^=kFc[1+0.001xFc^2^λ^3^/sin(2θ)]^-1/4^
0 restraints	Extinction coefficient: 0.0038 (11)

### Special details

**Table:**

Geometry. All e.s.d.'s (except the e.s.d. in the dihedral angle between two l.s. planes) are estimated using the full covariance matrix. The cell e.s.d.'s are taken into account individually in the estimation of e.s.d.'s in distances, angles and torsion angles; correlations between e.s.d.'s in cell parameters are only used when they are defined by crystal symmetry. An approximate (isotropic) treatment of cell e.s.d.'s is used for estimating e.s.d.'s involving l.s. planes.

### Fractional atomic coordinates and isotropic or equivalent isotropic displacement parameters (Å^2^)

**Table:**

	*x*	*y*	*z*	*U*_iso_*/*U*_eq_	
C1	−0.02574 (15)	0.14080 (13)	0.14955 (15)	0.0384 (4)	
H1	−0.0116	0.0650	0.1559	0.046*	
C2	−0.11433 (16)	0.19026 (13)	0.21703 (15)	0.0396 (4)	
C3	−0.13789 (17)	0.30501 (14)	0.21067 (18)	0.0479 (4)	
H3	−0.1979	0.3366	0.2601	0.057*	
C4	−0.07141 (17)	0.36854 (14)	0.13112 (18)	0.0479 (4)	
H4	−0.0867	0.4442	0.1242	0.058*	
C5	0.02062 (16)	0.31952 (12)	0.05933 (16)	0.0397 (4)	
C6	0.14059 (15)	0.18113 (12)	−0.00409 (15)	0.0369 (4)	
C7	0.20276 (17)	0.07630 (13)	0.00131 (16)	0.0423 (4)	
H7	0.2746	0.0694	−0.0433	0.051*	
C8	0.17220 (16)	0.28204 (13)	−0.05971 (15)	0.0377 (4)	
C9	0.27357 (16)	0.30700 (13)	−0.14343 (15)	0.0389 (4)	
C10	0.33469 (18)	0.40926 (14)	−0.12918 (18)	0.0490 (5)	
H10	0.3104	0.4595	−0.0668	0.059*	
C11	0.42963 (19)	0.43593 (15)	−0.20600 (19)	0.0547 (5)	
H11	0.4698	0.5036	−0.1945	0.066*	
C12	0.46660 (17)	0.36275 (15)	−0.30113 (17)	0.0467 (4)	
C13	0.40815 (17)	0.26118 (14)	−0.31569 (16)	0.0435 (4)	
H13	0.4330	0.2111	−0.3779	0.052*	
C14	0.31262 (17)	0.23437 (13)	−0.23732 (16)	0.0422 (4)	
H14	0.2738	0.1660	−0.2479	0.051*	
C15	0.5928 (2)	0.32902 (19)	−0.4794 (2)	0.0685 (6)	
H15A	0.6582	0.3633	−0.5241	0.103*	
H15B	0.6204	0.2588	−0.4449	0.103*	
H15C	0.5228	0.3180	−0.5394	0.103*	
N1	−0.18828 (14)	0.11943 (13)	0.29732 (14)	0.0482 (4)	
N2	0.04176 (12)	0.20585 (10)	0.07215 (12)	0.0356 (3)	
N3	0.09779 (14)	0.36523 (11)	−0.02137 (14)	0.0438 (4)	
O1	−0.17708 (14)	0.01936 (11)	0.28788 (14)	0.0623 (4)	
O2	−0.25834 (15)	0.16393 (13)	0.37021 (15)	0.0741 (5)	
O3	0.16791 (13)	−0.00450 (9)	0.06039 (13)	0.0548 (4)	
O4	0.55866 (13)	0.39871 (12)	−0.37535 (13)	0.0631 (4)	

### Atomic displacement parameters (Å^2^)

**Table:**

	*U*^11^	*U*^22^	*U*^33^	*U*^12^	*U*^13^	*U*^23^
C1	0.0429 (9)	0.0298 (7)	0.0421 (9)	−0.0019 (7)	−0.0011 (8)	0.0014 (7)
C2	0.0425 (10)	0.0377 (8)	0.0385 (9)	−0.0045 (7)	0.0019 (8)	−0.0010 (7)
C3	0.0515 (11)	0.0395 (9)	0.0532 (11)	0.0025 (8)	0.0086 (9)	−0.0072 (8)
C4	0.0546 (11)	0.0304 (8)	0.0594 (11)	0.0055 (8)	0.0083 (9)	−0.0021 (8)
C5	0.0467 (10)	0.0270 (7)	0.0448 (9)	0.0014 (7)	−0.0011 (8)	0.0011 (7)
C6	0.0396 (9)	0.0325 (8)	0.0385 (8)	−0.0008 (7)	0.0011 (7)	−0.0006 (7)
C7	0.0463 (10)	0.0350 (8)	0.0456 (10)	0.0034 (7)	0.0036 (8)	0.0010 (7)
C8	0.0432 (9)	0.0326 (8)	0.0370 (8)	0.0001 (7)	−0.0026 (7)	0.0012 (6)
C9	0.0436 (9)	0.0351 (8)	0.0378 (9)	−0.0004 (7)	−0.0009 (7)	0.0040 (7)
C10	0.0588 (12)	0.0380 (9)	0.0506 (10)	−0.0050 (8)	0.0070 (9)	−0.0049 (8)
C11	0.0641 (13)	0.0406 (9)	0.0603 (12)	−0.0170 (9)	0.0103 (10)	−0.0053 (8)
C12	0.0479 (10)	0.0484 (10)	0.0441 (10)	−0.0087 (8)	0.0035 (8)	0.0026 (8)
C13	0.0490 (11)	0.0428 (9)	0.0386 (9)	−0.0031 (8)	0.0015 (8)	−0.0022 (7)
C14	0.0504 (11)	0.0342 (8)	0.0418 (9)	−0.0074 (7)	−0.0004 (8)	0.0010 (7)
C15	0.0619 (14)	0.0834 (16)	0.0622 (13)	−0.0196 (12)	0.0228 (11)	−0.0136 (11)
N1	0.0485 (9)	0.0496 (9)	0.0465 (8)	−0.0048 (7)	0.0042 (7)	0.0000 (7)
N2	0.0399 (8)	0.0277 (6)	0.0388 (7)	0.0004 (5)	−0.0002 (6)	0.0000 (5)
N3	0.0514 (9)	0.0316 (7)	0.0487 (8)	0.0017 (6)	0.0044 (7)	0.0039 (6)
O1	0.0686 (10)	0.0432 (7)	0.0765 (10)	−0.0044 (6)	0.0153 (8)	0.0124 (7)
O2	0.0803 (11)	0.0707 (10)	0.0752 (10)	−0.0053 (8)	0.0386 (9)	−0.0078 (8)
O3	0.0638 (9)	0.0343 (6)	0.0676 (8)	0.0080 (6)	0.0155 (7)	0.0104 (6)
O4	0.0654 (9)	0.0664 (9)	0.0593 (8)	−0.0241 (7)	0.0201 (7)	−0.0072 (7)

### Geometric parameters (Å, º)

**Table:**

C1—C2	1.354 (2)	C9—C14	1.386 (2)
C1—N2	1.360 (2)	C9—C10	1.405 (2)
C1—H1	0.9300	C10—C11	1.370 (3)
C2—C3	1.409 (2)	C10—H10	0.9300
C2—N1	1.459 (2)	C11—C12	1.392 (3)
C3—C4	1.357 (2)	C11—H11	0.9300
C3—H3	0.9300	C12—O4	1.361 (2)
C4—C5	1.405 (2)	C12—C13	1.385 (2)
C4—H4	0.9300	C13—C14	1.385 (2)
C5—N3	1.330 (2)	C13—H13	0.9300
C5—N2	1.3961 (19)	C14—H14	0.9300
C6—N2	1.395 (2)	C15—O4	1.424 (2)
C6—C8	1.396 (2)	C15—H15A	0.9600
C6—C7	1.434 (2)	C15—H15B	0.9600
C7—O3	1.2197 (19)	C15—H15C	0.9600
C7—H7	0.9300	N1—O1	1.2184 (19)
C8—N3	1.360 (2)	N1—O2	1.220 (2)
C8—C9	1.464 (2)		
			
C2—C1—N2	117.71 (14)	C11—C10—H10	119.6
C2—C1—H1	121.1	C9—C10—H10	119.6
N2—C1—H1	121.1	C10—C11—C12	120.65 (17)
C1—C2—C3	122.79 (16)	C10—C11—H11	119.7
C1—C2—N1	117.32 (15)	C12—C11—H11	119.7
C3—C2—N1	119.88 (16)	O4—C12—C13	124.62 (17)
C4—C3—C2	118.83 (16)	O4—C12—C11	116.07 (16)
C4—C3—H3	120.6	C13—C12—C11	119.30 (17)
C2—C3—H3	120.6	C12—C13—C14	119.81 (16)
C3—C4—C5	119.67 (16)	C12—C13—H13	120.1
C3—C4—H4	120.2	C14—C13—H13	120.1
C5—C4—H4	120.2	C13—C14—C9	121.56 (15)
N3—C5—N2	111.14 (14)	C13—C14—H14	119.2
N3—C5—C4	129.99 (15)	C9—C14—H14	119.2
N2—C5—C4	118.85 (15)	O4—C15—H15A	109.5
N2—C6—C8	104.88 (13)	O4—C15—H15B	109.5
N2—C6—C7	122.83 (14)	H15A—C15—H15B	109.5
C8—C6—C7	131.31 (16)	O4—C15—H15C	109.5
O3—C7—C6	124.53 (17)	H15A—C15—H15C	109.5
O3—C7—H7	117.7	H15B—C15—H15C	109.5
C6—C7—H7	117.7	O1—N1—O2	123.60 (16)
N3—C8—C6	111.25 (15)	O1—N1—C2	118.41 (15)
N3—C8—C9	119.68 (14)	O2—N1—C2	118.00 (15)
C6—C8—C9	128.98 (15)	C1—N2—C6	131.33 (13)
C14—C9—C10	117.84 (16)	C1—N2—C5	122.11 (14)
C14—C9—C8	123.12 (15)	C6—N2—C5	106.55 (13)
C10—C9—C8	119.04 (15)	C5—N3—C8	106.16 (13)
C11—C10—C9	120.81 (17)	C12—O4—C15	117.45 (15)

### Hydrogen-bond geometry (Å, º)

**Table:**

*D*—H···*A*	*D*—H	H···*A*	*D*···*A*	*D*—H···*A*
C4—H4···N3^i^	0.93	2.53	3.412 (2)	158
C15—H15*A*···O3^ii^	0.96	2.52	3.423 (2)	158
C14—H14···O1^iii^	0.93	2.50	3.425 (2)	177

Symmetry codes: (i) −*x*, −*y*+1, −*z*; (ii) −*x*+1, *y*+1/2, −*z*−1/2; (iii) −*x*, −*y*, −*z*.
