# Supplementary figures and images for: Crystal structure of 2-(4-meth­oxy­phen­yl)-6-nitro­imidazo[1,2-a]pyridine-3-carbaldehyde
Source: Acta Crystallogr E Crystallogr Commun. 2015 Nov 21;71(Pt 12):o979–80. doi: 10.1107/S2056989015021957 (PMC4719933; doi:10.1107/S2056989015021957)

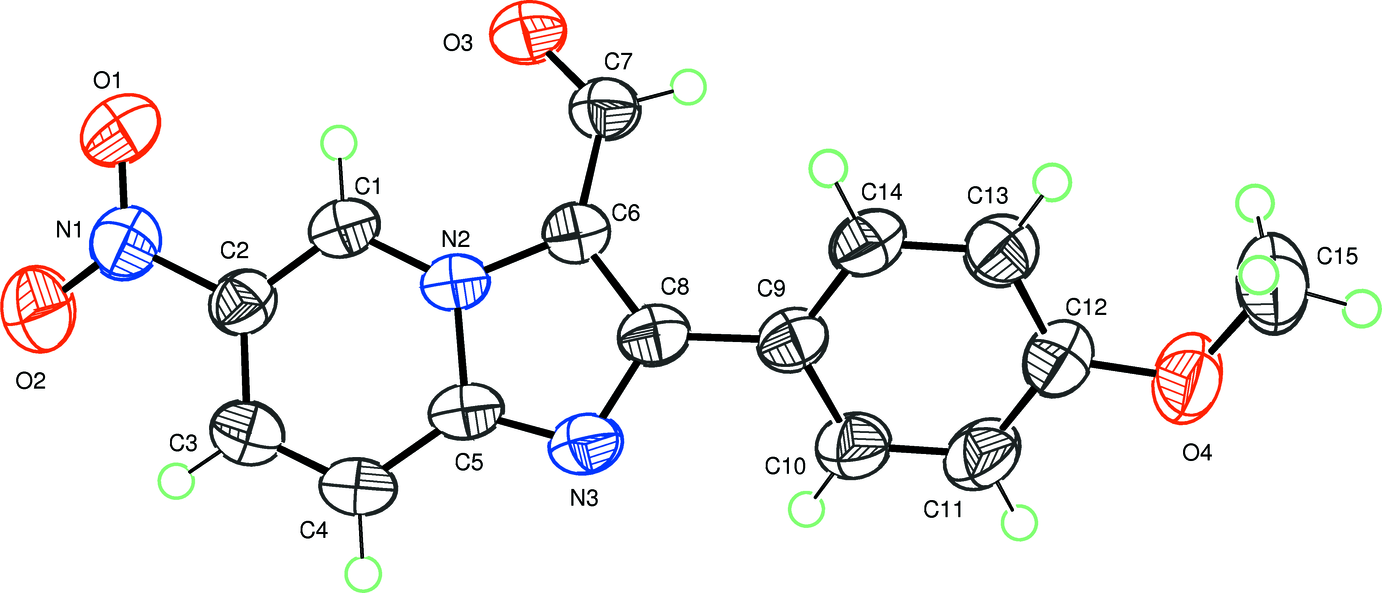

Supplement: Supplementary file 4 [file e-71-0o979-fig1.tif]

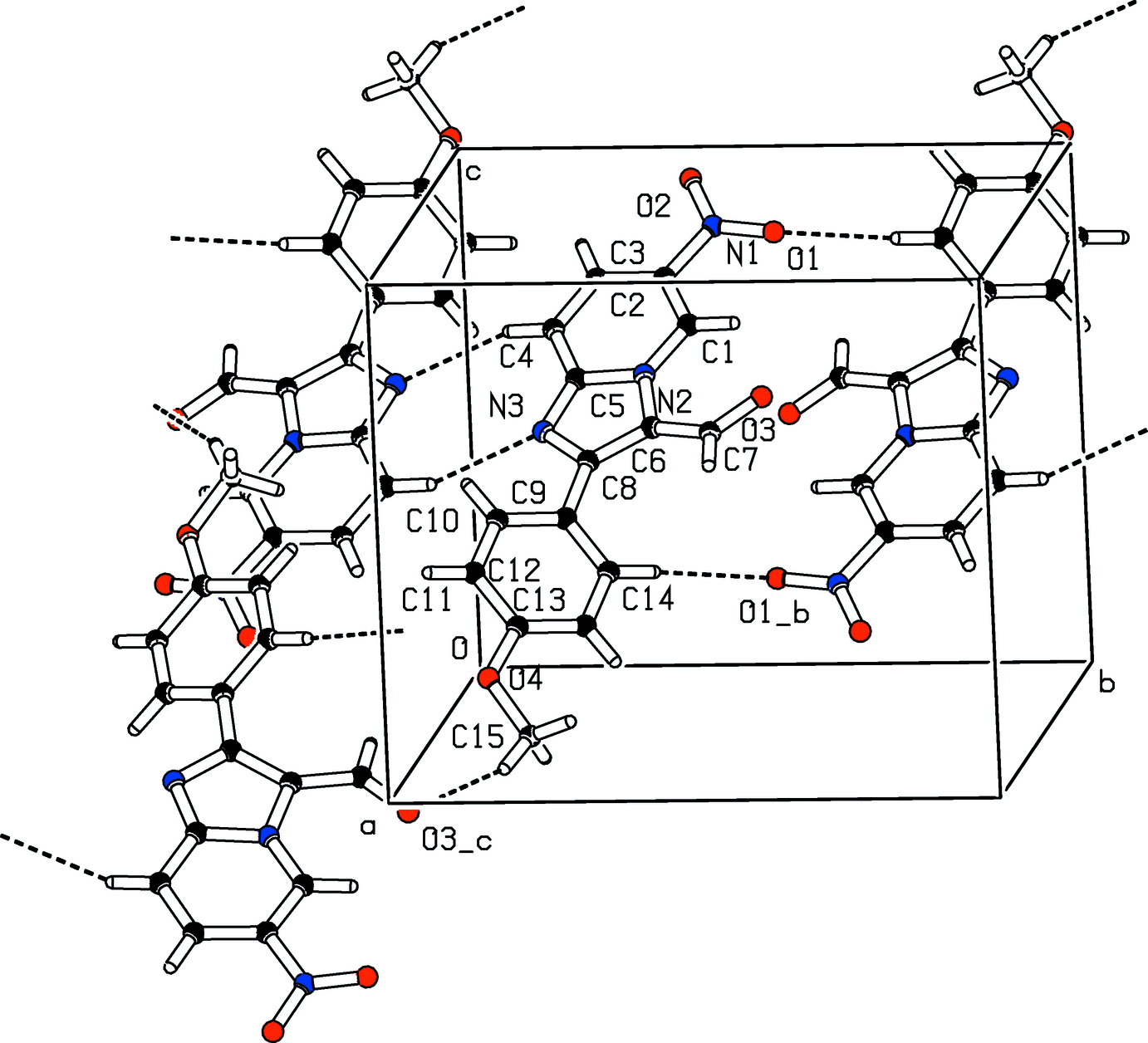

Supplement: Supplementary file 5 [file e-71-0o979-fig2.tif]
